# Reciprocal Clonal Dynamics of Independent *FLT3* D835V‐Positive Acute Myeloid Leukemia and Chronic Myeloid Leukemia With Gilteritinib

**DOI:** 10.1002/jha2.70289

**Published:** 2026-04-13

**Authors:** Kai Osone, Seiichiro Katagiri, SungGi Chi, Yosuke Minami, Akihiko Gotoh, Daigo Akahane

**Affiliations:** ^1^ Department of Hematology Tokyo Medical University Shinjuku Tokyo Japan; ^2^ Department of Hematology National Cancer Center Hospital East Kashiwa Chiba Japan

**Keywords:** acute myeloid leukemia, chronic myeloid leukemia, gilteritinib

## Abstract

We report a 65‐year‐old man with *FLT3* D835V‐mutated acute myeloid leukemia (AML) and *BCR::ABL1*‐positive chronic myeloid leukemia (CML) that coexisted as genetically independent clones. Cytogenetic and targeted sequencing analyses demonstrated that the AML and CML clones coexisted as distinct entities. During treatment with gilteritinib, the AML blasts regressed, and then the CML clone expanded. Subsequently, the CML burden declined as the AML clone regrew. This case highlights the importance of accurately assessing clonal changes using genetic analysis when implementing molecular targeted therapy for hematologic malignancies.

## 1 Introduction

Acute myeloid leukemia (AML) often develops from clonal hematopoiesis of indeterminate potential, in which stem cell clones with preexisting myeloid driver mutations proliferate and progress under selective pressure [1, 2, 3]. Among these, *FLT3* mutations (internal tandem duplication/tyrosine kinase domain [ITD/TKD]) act as driver lesions that confer a proliferative advantage and are key therapeutic targets for FLT3 inhibitors, such as gilteritinib [4]. In contrast, chronic myeloid leukemia (CML) is initiated and sustained by the *BCR::ABL1* fusion from the Philadelphia chromosome; *ABL1* kinase activity is necessary for pathogenesis and tractable to tyrosine‐kinase inhibition, producing durable molecular responses in chronic phase [5]. The co‐existence of CML with other neoplasms—particularly myeloproliferative neoplasms or chronic lymphocytic leukemia—has been reported [6, 7]. However, true concomitance of AML and CML as genetically independent diseases remains exceptionally rare. We present a 65‐year‐old man in whom AML and CML coexisted as independent clones at presentation, and whose subsequent course under FLT3 inhibition revealed reciprocal changes in clonal size.

## 2 Case Presentation

**FIGURE 1:**
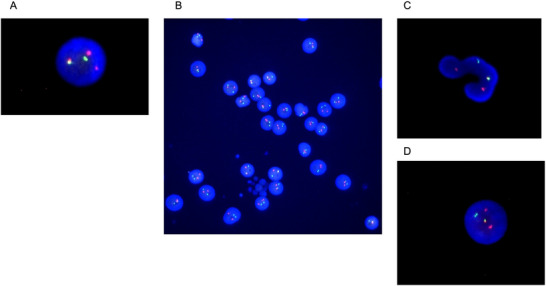
*BCR::ABL1* interphase FISH in a neutrophil‐enriched PB fraction (N‐FISH) and bone marrow *BCR::ABL1* FISH. (A) BM *BCR::ABL1* interphase FISH at diagnosis (overall positivity: 1.0% at diagnosis). Representative positive cells are shown. (B) Low‐magnification overview of Neutrophil‐FISH. As reliable discrimination between segmented neutrophil nuclei and mononuclear cells is limited at this magnification, Panel B is presented as an overview without cell‐type labeling. (C) Higher‐magnification image focusing on a morphologically segmented nucleus used for scoring. *BCR::ABL1* interphase FISH was performed using an Abbott (Vysis) dual‐color *BCR*/*ABL1* probe. *BCR* signals are shown in green and *ABL1* signals in red; *BCR::ABL1* fusion signals appear as yellow. (D) BM *BCR::ABL1* interphase FISH at Day 50 (overall positivity: 72% at Day 50). Representative positive cells are shown. BM, bone marrow; FISH, fluorescence in situ hybridization; N‐FISH, neutrophil‐FISH; PB, peripheral blood.

The patient was a 65‐year‐old man. Upon presentation, he had a fever, and his peripheral blood (PB) showed the following: white blood cell (WBC) count, 46 × 10^9^/L (95% blasts, 4% neutrophils, 0% eosinophils, 0% basophils, and 1% lymphocytes); hemoglobin concentration, 6.1 g/dL; platelet count, 39 × 10^9^/L. The bone marrow (BM) was hypercellular with 95% myeloblasts. Flow cytometry revealed myeloperoxidase‐negative blasts expressing CD13, CD33, CD34, and CD117, with partial HLA‐DR expression, consistent with AML with minimal differentiation. The conventional Q‐banding karyotype was 46, XY in 18 of 20 metaphases, with two of 20 being Philadelphia chromosomes. BM interphase fluorescence in situ hybridization (FISH) for *BCR::ABL1* revealed a positivity rate of 1.0% (Figure 1A). Molecular testing revealed a major *BCR::ABL1* load of 990 copies, as well as *FLT3*‐TKD positivity with a signal ratio of 1.02. The PB *BCR::ABL1* International Scale (IS)‐PCR result was 0.26% (control *ABL1* mRNA: 300,307 copies). Importantly, FISH analysis of mature neutrophil fractions separated by density‐gradient centrifugation, termed neutrophil‐FISH (N‐FISH) [8], revealed that *BCR::ABL1* was positive in mature granulocytes (segmented nuclei were 97% positive, while round nuclei were 0% positive) (Figure 1B,C), suggesting the presence of another chronic‐phase CML clone alongside AML.

Next‐generation sequencing performed in the Hematologic Malignancy‐SCREEN‐Japan‐02 study (UMIN000046371) [9] revealed *FLT3* D835V with a variant allele frequency (VAF) of 40.91%; *PHF6* G266R with a VAF of 90.5%; a *KMT2A* Exon 8–Exon 2 partial tandem duplication (*MLL*‐PTD) at 7201 copies; and *BCR::ABL1* at 29 copies. Overall, we concluded that the AML clone (*FLT3* D835V, *PHF6*, and *MLL*‐PTD) and the *BCR::ABL1*‐positive CML clone coexisted as distinct entities. This panel revealed no evidence of clonal hematopoiesis that could be considered the background for both clones.

**FIGURE 2:**
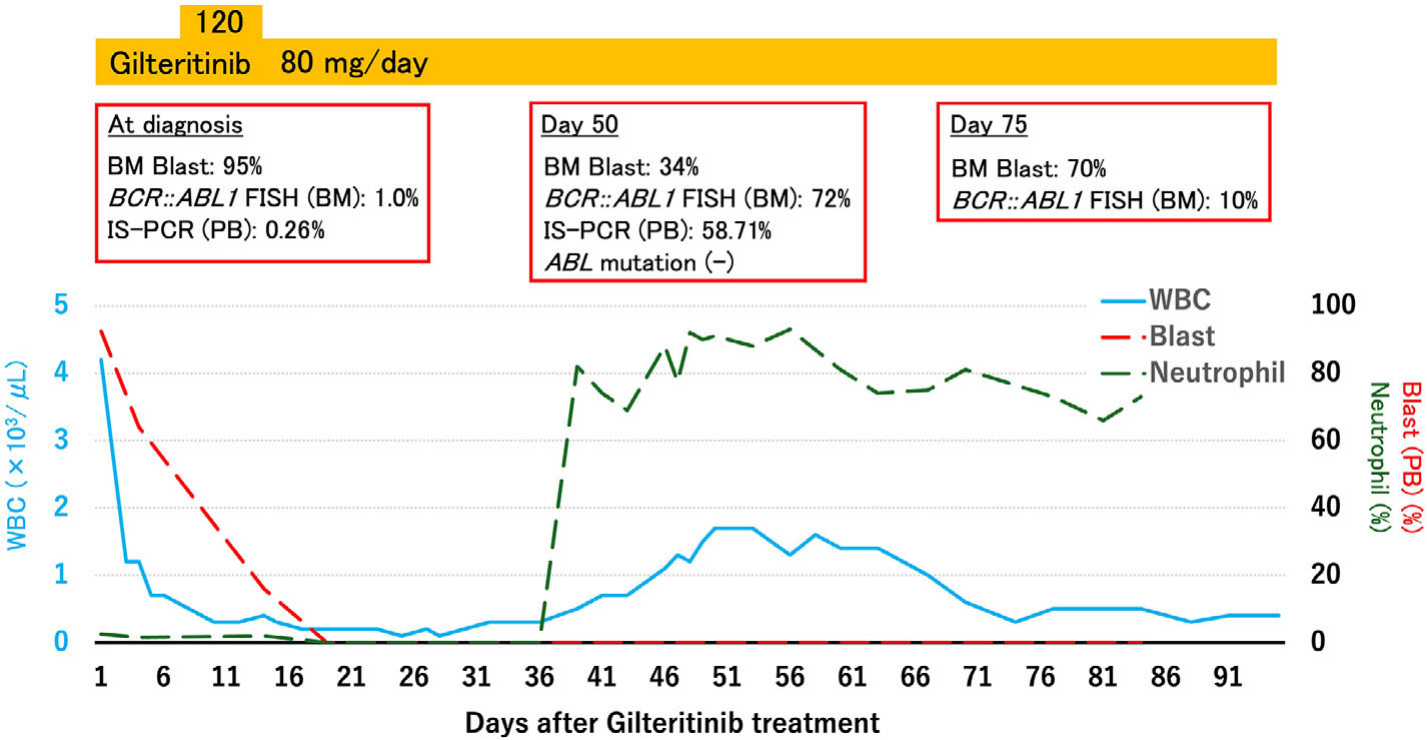
Clinical course after gilteritinib administration. The blue solid line indicates the PB WBC count, the green dashed line indicates the neutrophil percentage, and the red dashed line indicates the PB blast percentage. BM, bone marrow; FISH, fluorescence in situ hybridization; IS, *BCR::ABL1* International Scale; PB, peripheral blood; WBC, white blood cell.

Owing to severe pneumonia and pleurisy, the patient was deemed unable to undergo intensive chemotherapy. Cytoreduction was initiated with hydroxyurea and a 1‐week course of low‐dose cytarabine, followed by gilteritinib 120 mg/day (later reduced to 80 mg/day). By Day 50, the WBC count had recovered to 1.3 × 10^9^/L, with 0% myeloblasts and 80% neutrophils. At Day 50, the BM was hypocellular with 34% myeloblasts, and maturing neutrophils were evident. Moreover, BM *BCR::ABL1* FISH had increased to 72% (Figure 1D). N‐FISH showed that 100% of the segmented nuclei and 34% of the round nuclei were *BCR::ABL1* positive, indicating that the expanding circulating neutrophils were CML‐derived. Consistently, PB *BCR::ABL1* IS‐PCR increased to 58.71% (control *ABL1* mRNA: 41,894 copies). Owing to persistent infection and gastrointestinal bleeding, we were unable to escalate therapy and continued gilteritinib as a single agent. However, by Day 75, BM myeloblasts had re‐expanded to 70%, while BM FISH for *BCR::ABL1* had decreased to 10%. The patient died of progressive infection and leukemia (Figure 2).

## 3 Discussion

In this case, fluctuations in clone size were observed between AML and CML clones. Specifically, when *FLT3*‐mutant AML cells regressed under gilteritinib treatment, *BCR::ABL1*‐positive CML cells proliferated, as quantified by FISH and IS‐PCR. This clinical course is supported by two previous research reports. First, a recent genome‐wide reconstruction of CML evolution using whole‐genome sequencing of single‐cell‐derived colonies revealed that the *BCR::ABL1*‐driven clone grows explosively 3–14 years before diagnosis, exhibiting extremely high annual growth rates [10]. Crucially, most additional myeloid driver mutations are found in the *BCR::ABL1*‐negative (non‐Philadelphia) compartment, consistent with the existence of non‐Philadelphia clones within the same individual years before clinical detection.

Second, genomic analyses of Philadelphia‐negative AML that arose after tyrosine‐kinase inhibitor‐treated CML showed few or no shared somatic variants between the AML and antecedent CML [11]. This finding supports separate founding clones rather than linear transformation. It also supports the idea that therapy can reveal a preexisting, genetically independent myeloid clone. These data support the concept that distinct myeloid clones can coevolve in the same patient and that therapy can unmask a genetically independent clone.

In addition, the emergence or expansion of *BCR::ABL1*‐positive clones after FLT3 inhibitor treatment has been reported in *FLT3*‐mutant AML. Alotaibi et al. described three patients who experienced relapse after receiving FLT3 inhibitor regimens [12]. The researchers identified newly detectable *BCR::ABL1* fusion as a potentially targetable resistance mechanism in these patients. Moreover, Kim et al. reported the occurrence of p190 *BCR::ABL1* in patients with *FLT3*‐mutant AML who participated in a gilteritinib trial followed by re‐induction chemotherapy [13]. These reports suggest that suppression of an FLT3‐dependent compartment can favor expansion of ABL1‐dependent clones under therapeutic selection pressure. Our observation of clonal replacement between *FLT3*‐mutant AML and *BCR::ABL1*‐positive CML under gilteritinib is consistent with this concept, although in our patient, the CML clone became quantitatively dominant as AML regressed, rather than emerging as *BCR::ABL1*‐positive AML.

The main limitation of this study is that it was a single‐patient observation without clone‐resolved sequencing at each timepoint. Nonetheless, time‐dependent changes in neutrophil *BCR::ABL1* FISH and IS‐PCR, together with targeted sequencing data, strongly support the presence of competing coexisting clones with differential sensitivity to FLT3 inhibition.

In summary, our case provides lineage‐resolved, time‐resolved evidence that AML and CML can coexist as independent clones and undergo reciprocal clonal evolution under FLT3 inhibitor treatment. This case demonstrates the importance of accurately assessing clonal changes using genetic analysis and implementing molecular targeted therapy for hematologic malignancies.

## Author Contributions

K.O., S.K., A.G., and D.A. treated the patient. S.C. and Y.M. performed the NGS analyses in the HM‐SCREEN‐JAPAN 02 studies. K.O. and S.K. designed the study and wrote the manuscript. D.A. and A.G. supervised the project. All authors contributed to the data analyses and drafting of the manuscript.

## Funding

The authors have nothing to report.

## Ethics Statement

This study was conducted in accordance with the principles of the Declaration of Helsinki. This study was reviewed and approved by the ethics committee of Tokyo Medical University Hospital.

## Consent

Written informed consent for publication was obtained from the patient.

## Conflicts of Interest

Yosuke Minami reports receiving honoraria from Pfizer, Bristol‐Myers Squibb, Novartis Pharma, Daiichi Sankyo, and Astellas Pharma; and receiving research funding from Bristol‐Myers Squibb, Chugai Pharmaceutical, Novartis Pharma, Takeda Pharmaceutical, Ono Pharmaceutical, and CMIC. Akihiko Gotoh reports receiving honoraria from Novartis Pharma and Alexion Pharma; and receiving research funding from Ono Pharmaceutical, Takeda Pharmaceutical, Nippon Shinyaku, and Chugai Pharmaceutical. The other authors declare no conflicts of interest.

## Data Availability

The data that support the findings of this study are available from the corresponding author upon reasonable request.
